# Intratumoral immune activation with TLR4 agonist synergizes with effector T cells to eradicate established murine tumors

**DOI:** 10.1038/s41541-020-0201-x

**Published:** 2020-06-16

**Authors:** Tina C. Albershardt, Jardin Leleux, Andrea J. Parsons, Jordan E. Krull, Peter Berglund, Jan ter Meulen

**Affiliations:** Immune Design, a wholly-owned subsidiary of Merck & Co., Inc, Kenilworth, NJ USA

**Keywords:** Vaccines, Tumour immunology

## Abstract

Effective T cell-based immunotherapy of solid malignancies requires intratumoral activity of cytotoxic T cells and induction of protective immune memory. A major obstacle to intratumoral trafficking and activation of vaccine-primed or adoptively transferred tumor-specific T cells is the immunosuppressive tumor microenvironment (TME), which currently limits the efficacy of both anti-tumor vaccines and adoptive cell therapy (ACT). Combination treatments to overcome TME-mediated immunosuppression are therefore urgently needed. We combined intratumoral administration of the synthetic toll-like receptor 4 agonist glucopyranosyl lipid A (oil-in-water formulation, G100) with either active vaccination or adoptive transfer of tumor-specific CD8 T cells to mice bearing established melanomas or orthotopically inoculated glioblastomas. In combination with cancer vaccines or ACT, G100 significantly increased expression of innate immune genes, infiltration and expansion of activated effector T cells, antigen spreading, and durable immune responses. Complete tumor regression of both injected and non-injected tumors was observed only in mice receiving combination immunotherapy. TLR4-based intratumoral immune activation may be a viable approach to enhance the efficacy of therapeutic cancer vaccines and ACT in patients.

## Introduction

Immune checkpoint blockade and adoptive T cell therapy have shown impressive clinical results and solidified immunotherapy as a new pillar of cancer therapy^1^. However, the majority of cancer patients to date do not benefit from immune checkpoint inhibitors. Adoptive cell therapy (ACT) has generally not been successfully applied to patients with solid tumors, and cancer vaccines have largely failed to deliver meaningful clinical benefit. Interestingly, the single most predictive success factor of any immunotherapy is the presence of a T cell-inflamed tumor microenvironment (TME), as shown by a large number of clinical studies in which pre-treatment immune status of the TME was correlated with clinical response^2,3^. Preclinically, it has been shown that eradication of aggressive murine B16 melanomas requires activated, non-exhausted effector T cells to traffic to the TME, which can be achieved by vaccination with a lentiviral vector encoding a tumor antigen, or transfer of activated tumor-specific T cells, followed by combined intratumoral injections of toll-like receptor 3 and 9 agonists^4^. Another approach used a complex 4-component combination immunotherapy consisting of a lymph node-targeted peptide vaccine, an anti-tumor antibody, a checkpoint inhibitor, and recombinant IL-2^5^. And most recently, an optimal dosing of an agonist of stimulator of interferon genes, combined with two checkpoint inhibitors, was shown to eradicate treated tumors and generate durable anti-tumor responses that rejected subsequent tumor re-challenges in majority of the cured mice^6^. These regimens are currently the only cancer vaccine regimens capable of eradicating notoriously difficult-to-treat, large B16 melanoma tumors through engagement of both innate and adaptive immune responses.

Activation of local and systemic immune responses through intratumoral injection of the synthetic toll-like receptor 4 (TLR4) agonist glucopyranosyl lipid A (GLA) is a therapeutic approach currently being investigated in the clinic in injectable solid and hematological malignancies^7^. GLA, a synthetic derivative of the lipid A tail of lipopolysaccharides, when formulated in a stable oil-in-water emulsion (SE; i.e., G100 is GLA formulated in SE), has been shown preclinically to activate macrophages and dendritic cells and to induce the major T cell homing chemokines (e.g., CXCL9 and CXCL10^8^) in a TLR4-dependent manner^9–12^. It promotes Th1-type inflammatory changes at locally injected sites and systemic T cell responses in patients with clinical activity, and a complete response has been reported in a Merkel cell carcinoma patient^13^. Here, we combined intratumoral immune activation using G100 with either active vaccination with a dendritic cell-targeting lentiviral vector (ZVex®) or adoptive transfer of tumor-specific T cells to increase T cell trafficking to the tumor and sustain immune cell functions. Direct expression of tumor antigens in dendritic cells with ZVex is highly effective in priming CD8 T cells in preclinical models^14,15^ and has resulted in immunological and clinical responses in patients, including one near-complete response in a sarcoma patient^16^. In this study, we show that G100 synergized with both ZVex immunization and ACT in aggressive murine tumor models, supporting the evaluation of these immunotherapeutic combinations in the clinic.

## Results

### G100 promotes a T cell-inflamed TME

To determine shifts in the population of immune cells post-G100 treatment, B16 tumors were harvested 24 h after the last of four G100 treatments, and single cell suspensions were then stained for cell surface markers and analyzed by flow cytometry (Supplementary Fig. 1). G100 led to an overall increase in infiltration of effector cells (Supplementary Fig. 1a), including T cells and NK cells; immune-activating myeloid cells (Supplementary Fig. 1b), including macrophages and CD103^+^ CD11c^+^ tumor-residing dendritic cells; and CD103^+^ CD8^+^ tissue-resident memory cells. G100 did not significantly alter presence of immunosuppressive Ly6C^+^ Ly6G^−^ or Ly6C^+^ Ly6G^+^ myeloid cells in the tumor.

### G100–ZVex combination eradicates established tumors in murine melanoma and glioblastoma models

**Fig. 1:**
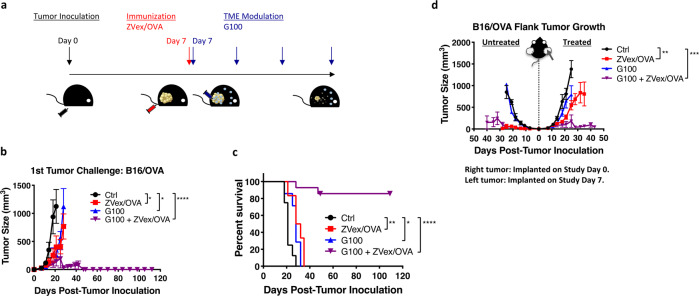
G100–ZVex combination eradicates established tumors. **a** Schematic of tumor inoculation, ZVex immunization, and G100 administration. Female C57BL/6 mice (*n* = 18/group) were inoculated with B16/OVA cells (flank, subcutaneously) on day 0. Once tumors became palpable (averaged 5 mm in diameter), mice were immunized once with ZVex/OVA and given intratumoral G100 that was continued twice weekly until observation of complete tumor regression or euthanized due to tumor burden. **b** Tumor size and **c** survival were monitored and recorded at least 2–3 times weekly. **d** In a bilateral B16/OVA model, tumor inoculation of the treated tumor and its treatment regimen were the same as described in (**a**) (*n* = 9/group). Inoculation of the vehicle-treated tumors occurred on day 7. Tumor sizes were plotted as average tumor volume ± SEM. Death was recorded as a result of death defined by euthanasia criteria. Data are representative of at least three independent experiments (*p < 0.05, **p < 0.01, ***p < 0.001, ****p < 0.0001).

Mice bearing B16 melanoma cells expressing ovalbumin (B16/OVA) were treated with G100, ZVex encoding ovalbumin (ZVex/OVA), or the G100–ZVex combination (Fig. 1a). By day 21, mice treated with G100 or ZVex/OVA alone exhibited significantly delayed tumor growth compared to control animals (Fig. 1b), which translated to significant survival benefit (Fig. 1c). The G100–ZVex combination induced complete tumor regression in 16/18 (88.9%) mice (Fig. 1b), leading to highly significant long-term survival with no tumor recurrence over at least 120 days (Fig. 1c). Similar therapeutic responses were observed in an orthotopic murine model of GL261 glioblastoma expressing ovalbumin and luciferase (GL261/OVA), where tumor growth was monitored by bioluminescent imaging (Supplementary Fig. 2). By day 30, while three intratumoral injections of G100 or a single immunization with ZVex/OVA only slowed tumor growth compared to vehicle-treated controls, the G100–ZVex combination induced complete tumor regression in 2/3 (66.7%) mice.

To model a metastatic setting in which not all tumor lesions could be injected, mice were inoculated with B16/OVA cells in the right flank, followed by inoculation in the left flank a week later. Tumor-bearing mice were then treated with G100, ZVex/OVA, or the G100–ZVex combination (Fig. 1d). G100 was administered only to the tumor on the right flank. The tumor on the left flank was not given intratumoral G100 in order to evaluate the effect of systemically induced immune responses on non-injectable tumors. By day 21, G100 monotherapy had minimal effect on growth of either treated or untreated tumors, whereas ZVex monotherapy delayed tumor growth in the right flank and tumor regression in the left. Strikingly, the G100–ZVex combination induced complete regression of both tumors in 5/9 (55.6%) mice.

### Anti-tumor efficacy of G100–ZVex combination is CD8 T cell-dependent

**Fig. 2:**
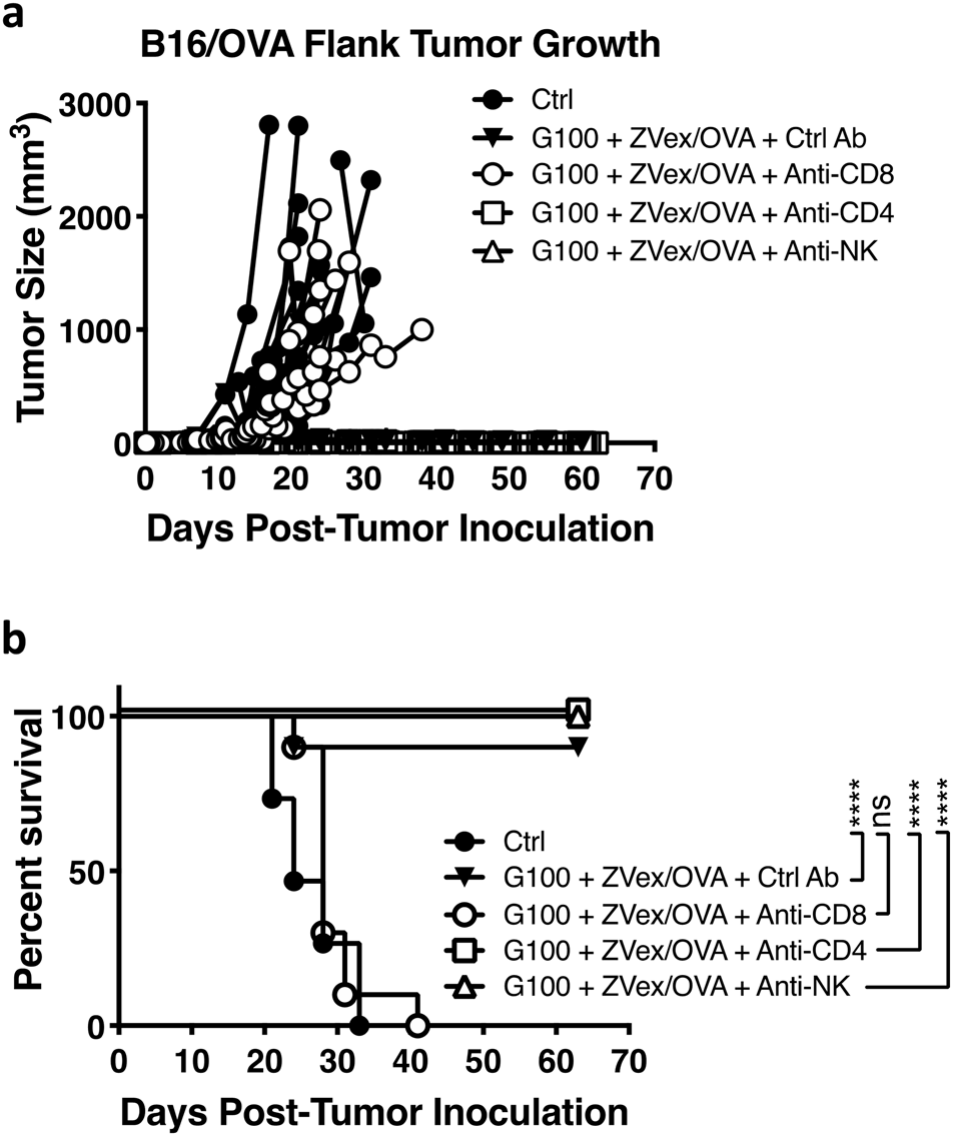
Anti-tumor efficacy induced by G100–ZVex combination is dependent on CD8 T cells. Female C57BL/6 mice (*n* = 10–15/group) were inoculated with B16/OVA cells (flank, subcutaneously) on day 0. On day 4, mice were given intraperitoneal administration of depletion antibodies as labeled in the figure. Once tumors became palpable (averaged 5 mm in diameter), mice were immunized once with ZVex/OVA and administered intratumoral G100 that was continued twice weekly until observation of complete tumor regression or euthanized due to tumor burden. **a** Tumor size and **b** survival were monitored and recorded at least 2–3 times weekly. Tumor sizes were plotted as average tumor volume ± SEM. Death was recorded as a result of death defined by euthanasia criteria. Data are representative of at least two independent experiments (****p < 0.0001).

To identify cell populations mediating anti-tumor control induced by the G100–ZVex combination, depletion of CD8 T cells, CD4 T cells, or NK cells was performed in mice post-tumor inoculation but pre-ZVex immunization and/or G100 administration. Tumor-bearing mice with depleted CD8 T cells failed to respond to the combination therapy, with tumor growth (Fig. 2a) and survival rate (Fig. 2b) similar to vehicle-treated tumor-bearing mice. In contrast, depletion of CD4 T cells or NK cells had minimal effect on G100–ZVex-mediated anti-tumor efficacy, showing that CD8 T cells are the main effectors induced by the combination therapy.

### G100–ZVex combination inflames the TME, increases T cell fraction and clonality, and induces antigen spreading

In numerous tumor types, improved clinical outcomes are typically associated with a T cell-inflamed TME, characterized by increased infiltration of lymphocytes and increased ratio of CD8 T cells to regulatory T cells^2,3^. Changes in the TME of B16/OVA-bearing mice treated with G100-, ZVex-, or the G100–ZVex combination were evaluated using gene expression profiling and flow cytometry analysis. Tumors were harvested 24 h after the last of four G100 treatments and 12 days post-ZVex immunization (to capture peak levels of ZVex-induced antigen-specific CD8 T cells, Supplementary Fig. 3). Expression levels of RNA isolated from tumors were determined using the PanCancer Immune Profiling Panel from Nanostring Technologies, which evaluated 770 immune response-related gene expression. The G100–ZVex combination highly increased gene expression of innate and adaptive immune pathways in all mice: dendritic cell, macrophage, NK cell, T cell, and B cell functions; cytokines and receptors (Supplementary Fig. 4a), including trafficking molecules such as CXCR3, CXCL9, CXCL10, and CXCL11 (Supplementary Fig. 4b). ZVex monotherapy induced much narrower and lower gene expression changes, with G100 monotherapy inducing the lowest gene expression changes.

Mice immunized with ZVex/OVA developed OVA-specific CD8 T cells, with responses peaking around 10 days post-immunization (Supplementary Fig. 3). To evaluate how treatments affect tumor infiltration of ZVex-induced OVA-specific effector T cells, tumor-infiltrating lymphocytes (TILs) were stained for flow cytometry analysis (Supplementary Fig. 5). In the TME of mice treated with G100 alone, percent TILs increased only marginally (1.1-fold), compared to controls (Supplementary Fig. 5a, top panel, and 5b). In contrast, mice treated with ZVex alone had a 1.8-fold increase in percent TILs (Supplementary Fig. 5a, top panel, and 5b) and a 18.4-fold increase in OVA-specific CD8 T cells (Supplementary Fig. 5a, bottom panel, and 5b). The G100–ZVex combination led to a 4.2-fold increase in percent TILs and a 28.8-fold increase in OVA-specific CD8 T cells. In both ZVex- and G100–ZVex-treated mice, the ratio of tumor-specific CD8 T cells to regulatory T cells in the tumor was increased by a factor of more than 30 (Supplementary Fig. 5c). Consistent with these observations, TCR analysis revealed that tumors from mice treated with ZVex and the G100–ZVex combination had increased T cell fraction, and the infiltrating T cells had increased clonality (Supplementary Fig. 6).

### G100–ZVex combination induces durable tumor-specific immune responses

**Fig. 3:**
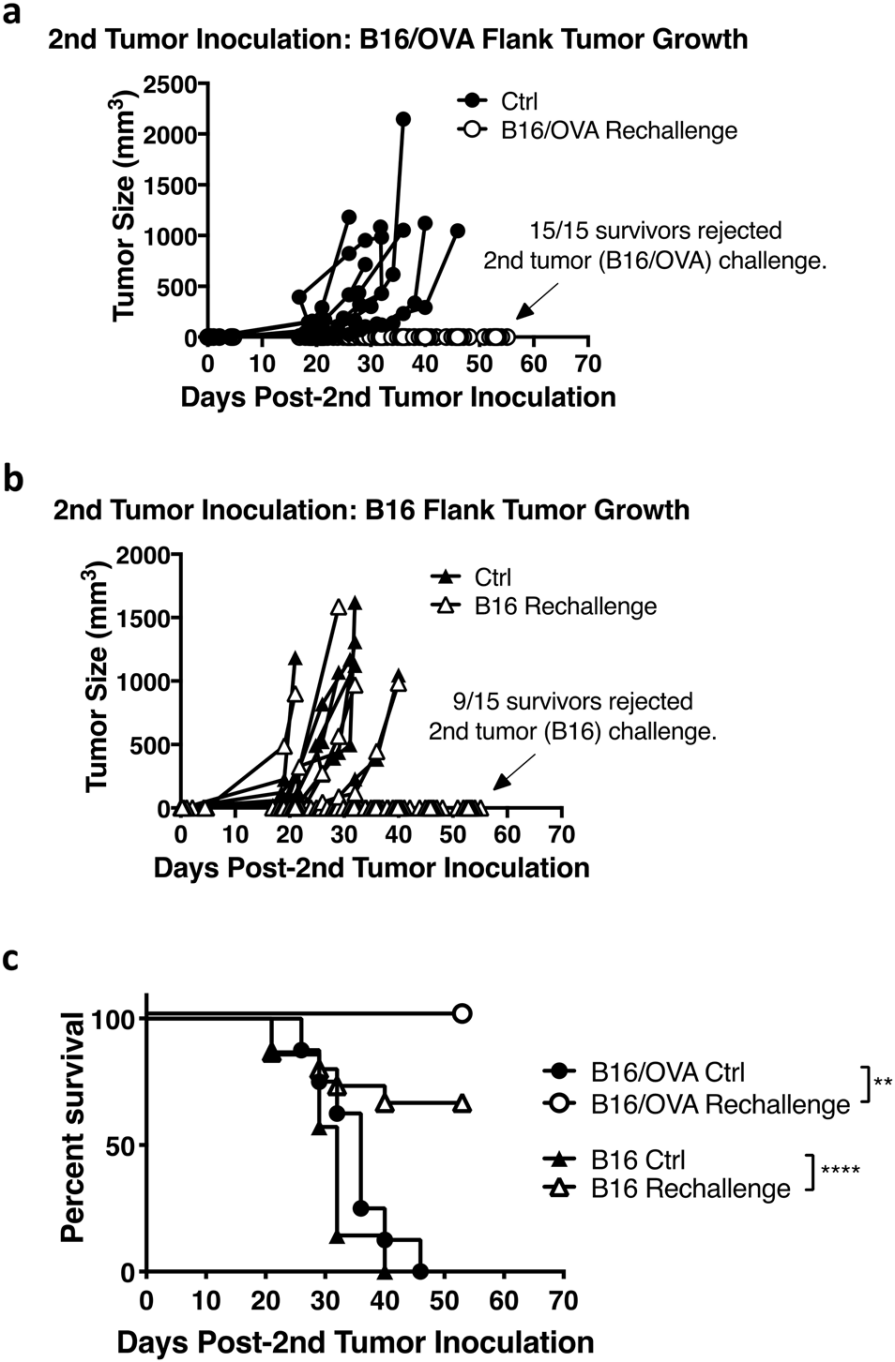
G100–ZVex combination induces durable anti-tumor immune responses. Female C57BL/6 mice (*n* = 10–16/group) treated with the G100–ZVex combination and had complete tumor regression were re-challenged with **a** B16/OVA or **b** B16 cells, inoculated in the opposite flank of the original tumor challenge. Tumor size and **c** survival were monitored and recorded at least 2–3 times weekly. “Ctrl” group of mice were age-matched naïve mice, inoculated with **a** B16/OVA or **b** B16 cells. Tumor sizes were plotted as average tumor volume ± SEM. Death was recorded as a result of death defined by euthanasia criteria. Data are representative of at least 2 independent experiments (**p < 0.01, ****p < 0.0001).

To evaluate successful generation of tumor-specific immunological memory, G100–ZVex-treated mice with completely regressed B16/OVA tumors (“survivors”) were given a second inoculation of either the original tumor cells (B16/OVA, Fig. 3a) or parental B16 cells that did not express OVA (Fig. 3b). All survivors rejected a second inoculation with B16/OVA (Fig. 3a), with 100% survival (Fig. 3c). When survivors were inoculated with the wildtype parental B16 cell line instead, 9/15 (60%) of mice were also able to reject the B16 challenge (Fig. 3b), suggesting that the G100–ZVex combination induced B16-specific immune responses through antigen spreading that could be recalled. To confirm the development of antigen spreading, splenic lymphocytes were isolated from survivors and evaluated for the presence of B16-specific CD8 T cells (Supplementary Fig. 7). Isolated splenocytes were stimulated with mTRP1_181–188_ or mTRP2_180–188_ (i.e., H-2b-restricted immunogenic epitopes of melanoma antigens) in mixed lymphocyte cultures (MLCs) for 5 days to allow selective proliferation of the respective antigen-specific CD8 T cells and thus increase the chance of their detection via multimer staining. Lymphocytes stimulated with an irrelevant peptide defined the basal level of antigen-specific CD8 T cells present in culture after the 5-day MLCs. Only lymphocytes from survivors developed significant levels of mTRP1-specific (Supplementary Fig. 7a, *p* = 0.0184) and mTRP2-specific (Supplementary Fig. 7b, *p* = 0.44) CD8 T cells, above background. These findings support that the G100–ZVex combination induced antigen spreading.

### G100 enhances anti-tumor efficacy of adoptively transferred OT-1 or PMEL T cells in B16 model

**Fig. 4:**
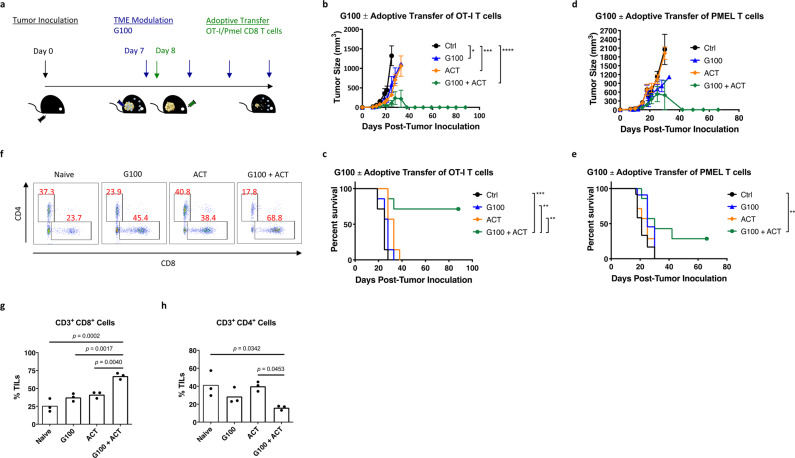
G100 enhances adoptive cell therapy. **a** Schematic of tumor inoculation, adoptive transfer, and G100 administration. Female C57BL/6 mice (*n* = 8–10/group) were inoculated with B16/OVA or B16 cells (flank, subcutaneously) on Day 0. Once tumors became palpable (averaged 5 mm in diameter), tumor-bearing mice received adoptive transfer of CD8 T cells isolated from OT-I or PMEL mice, respectively, and given intratumoral G100 that was continued twice weekly until observation of complete tumor regression or euthanized due to tumor burden. **b** Tumor size and **c** survival plots for mice inoculated with B16/OVA cells and received adoptive transfers of OT-I T cells. **d** Tumor size and **e** survival plots for mice inoculated with B16 cells and received adoptive transfers of PMEL T cells. Tumor sizes were plotted as average tumor volume ± SEM. Death was recorded as a result of natural death and death defined by euthanasia criteria. **f** Representative flow cytometry diagrams of tumor-infiltrating T cells, collected 3 days post-ACT and two G100 injections, with average percent CD8 and CD4 T cells of total CD3 T cells per group indicated, respectively, within the diagrams. Individual percent **g** CD8 and **h** CD4 T cells of total CD3 T cells per mouse per group are graphed. An average of 2 × 10^6^ events per sample were collected for analysis. Gating strategy: Singlets (SSC-A = SSC-H), lymphocytes (FSC-A vs. SSC-A), live cells (fixable live/dead stain negative), T cells (CD3^+^ B220^−^), CD8 T cells (CD8^+^ CD4^−^) and CD4 T cells (CD8^−^ CD4^+^).Data are representative of at least two independent experiments (**p < 0.01, ***p < 0.001).

Because G100 enhanced the therapeutic efficacy of ZVex-induced T cells via inflammation of the TME resulting in increased trafficking of ZVex-induced tumor-specific CD8 T cells, we hypothesized that G100 could also combine synergistically with ACT via a similar mechanism. To assess the therapeutic potential of the G100–ACT combination, once tumors became palpable, CD8 T cells isolated from OT-I or PMEL transgenic mice were adoptively transferred to B16/OVA- or B16-bearing mice, respectively (Fig. 4a). G100 was administered intratumorally every 2–3 days until tumors completely regressed or until mice were euthanized due to tumor burden. Mice that received the G100–ACT combination had significantly enhanced tumor protection compared to mice that received G100 or ACT alone. In the OVA-targeting ACT model, the G100–ACT combination resulted in complete tumor regression in 5/7 mice (Fig. 4b), conferring highly significant long-term survival (Fig. 4c). Similar synergy between G100 and ACT was also observed in the PMEL-targeting model, with the G100–ACT combination inducing complete tumor regression in 2/7 mice (Fig. 4d) and significant survival benefit (Fig. 4e). In both models, monotherapy with either G100 or ACT at best delayed tumor growth with minimal improvement in survival, compared to controls.

To track the trafficking of transferred T cells, we labeled CD8 T cells isolated from OT-I mice (CD45.2^+^) with CFSE and transferred these cells into congenic B6.SJL mice (CD45.1^+^) bearing B16/OVA tumors (Supplementary Fig. 8a). Cells were isolated from tumors, spleens, tumor-draining lymph nodes (TDLNs), and non-tumor-draining lymph nodes (NTDLNs) 24 h post-G100 treatment and stained for flow cytometry analysis. As expected, more than 95% of the transferred CD8 T cells (CD45.2^+^, CFSE^+^) were OVA-specific (Supplementary Fig. 8b) and found in all harvested tissues (Supplementary Fig. 8c). However, the proliferation of transferred cells was observed only in tumors and TDLNs, visualized by dilution of the CFSE signal (Supplementary Fig. 8c). Consistent with these observations, while G100 or ACT alone increased CD8 T cell infiltration of B16 tumors (Fig. 4f, g), tumors from G100–ACT-treated B16-bearing mice had the greatest percentage of tumor-infiltrating CD8 T cells. Although ACT alone minimally affected the percentage of CD4 T cells within the TME, G100 alone and in combination with ACT decreased CD4 T cell infiltration of B16 tumors (Fig. 4f, h), suggesting G100 could potentially be driving a decrease in infiltrating regulatory T cells. These findings demonstrate that modulation of the TME with G100 dramatically enhanced ACT-mediated anti-tumor control by increasing tumor-infiltrating effector cells and potentially decreasing regulatory T cells.

## Discussion

In preclinical models of solid tumors, especially B16 melanoma, the eradication of established tumors through therapeutic vaccination is notoriously difficult to achieve. Some of the earliest approaches to modify the TME date back to the early 1900s, when W. Coley showed that injection of live bacteria or bacterial extracts, which are now known to contain TLR 2,TLR 4, and TLR9 agonists, resulted in the regression of solid tumors^17^. Other critical factors have been reviewed^18,19^ and summarized in the “cancer-immunity cycle” as cancer cell death, cancer antigen presentation, priming, and activation of antigen-presenting cells and T cells, trafficking of T cells to the tumor, and tumor infiltration, recognition and killing of cancer cells^20^. In this context, the “cancer-immune set point” defines the inherent immunological status of a patient, with the immunosuppressive TME of most solid tumors representing a major obstacle to effective T cell-based immunotherapies^21,22^.

Using cancer vaccines or adoptively transferred T cells, we demonstrate here that the tumor-killing efficacy of CD8 T cells against established melanoma and glioblastoma tumors can be greatly enhanced by modulating the TME with a potent synthetic TLR4 agonist. This resulted not only in local but also anenestic responses and prevented tumor recurrence through the generation of tumor-specific immunological memory. Additionally, induction of antigen spreading by the G100–ZVex combination may have contributed to the generation of effective and durable anti-tumor immune responses. While we propose the presence of melanoma-specific T cells in survivor mice, who were not previously immunized against those antigens, is evidence of vaccine-induced antigen spreading (Supplementary Fig. 7), we recognize a caveat of the ex vivo culture system used for assessment: to improve detection of these melanoma-specific T cells, ex vivo 5-day MLCs were required. Lack of antigen-specific T cells detected in MLCs from naïve murine splenocytes ruled out de novo generation of antigen-specific T cells during this 5-day period and in turn suggested MLCs largely allowed proliferation of antigen-specific T cells that were already present in survivor splenocytes. While the data promisingly support vaccine-induced antigen spreading, it is difficult to translate significance of an ex vivo expanded culture to meaningful biological impact in vivo. Lastly, the model system used in these studies would not distinguish between immunological memory generated by the G100–ZVex combination or simply from exposure to the tumor alone. However, we have previously demonstrated that untreated B16-bearing mice did not generate detectable levels of tumor-specific T cells (unpublished observations), in support that protective immunological memory demonstrated in this report is likely due to the combination.

T cell-inflamed tumors respond better to immunotherapy than non-T cell-inflamed tumors^2,3^. Mice treated with ZVex or G100 alone had significantly delayed tumor growth but no regression (Fig. 1). While ZVex-induced T cells were able to infiltrate the tumor (Supplementary Fig. 5a) and G100-treated tumors were moderately more inflamed than vehicle-treated tumors (Supplementary Fig. 4a), it was only the combination of these two therapeutic platforms that resulted in complete tumor regression (Fig. 1). The TME of mice treated with the G100–ZVex combination were significantly more permissive of immune cell functions than either treatment alone. These findings demonstrate that to achieve effective immunotherapy, innate immune responses in the TME need to be activated, likely so that the proper chemokines could be produced to support and sustain the infiltration of effector cells, especially effector CD8 T cells (Fig. 2). Hence, modulation of the TME with G100 also greatly enhanced ACT-mediated anti-tumor control by increasing tumor-infiltrating CD8 T cells (Fig. 4).

To overcome immune escape due to heterogenous expression of antigens in primary tumors and metastases, immune responses against multiple tumor antigens must be induced. Successful approaches have been reported in both preclinical^23,24^ and clinical settings^25–27^ and are based on direct targeting of multiple tumor-associated antigens^23,25^ or the use of tumor cell lysates, which in theory would include all available tumor antigens^28–30^. Targeting of tumor antigens requires their identification and characterization and is limited by expression capacity of the vaccine vector and epitope immunodominance phenomena^31,32^. The use of tumor cell lysates on the other hand is hampered by the weak or absent immunogenicity of their antigens due to generally very low abundance^33^. Induction of antigen spreading has been associated with improved clinical outcome and may be the only feasible option to control immune escape of tumors^34^. In our models, the only treatment regimen to most successfully eradicate established B16/OVA tumors was the G100–ZVex combination. Tumor-bearing mice immunized against one model antigen (OVA), in combination with repeated intratumoral G100 treatments, developed effector T cells not only against the targeted antigen (OVA) but also against at least two non-targeted other B16 melanoma antigens (mTRP1 and mTRP2) (Supplementary Fig. 7). The G100–ZVex combination likely induced this observed antigen spreading through the synergy achieved by the combination: (1) ZVex induced a strong effector T cell response against a tumor antigen, resulting in initial tumor-killing and subsequent release of additional tumor antigens; and (2) together with intratumoral G100, the combination highly activated immune cells within the TME, allowing the released tumor antigens to be efficiently processed and presented by antigen-presenting cells to induce T cell responses against the released tumor antigens. To model the effect of antigen spreading on control of tumors that have decreased expression of targeted antigen(s), we inoculated mice with B16/OVA cells, immunized the mice against OVA with the G100–ZVex combination regimen, and re-challenged survivors with parental B16 cells (i.e., tumors with “loss” of OVA expression). Of the mice that were cured of the original B16/OVA tumor, all were able to reject a second B16/OVA challenge (Fig. 3). While protection against the parental B16 re-challenge was 60%, this finding is promising, as it suggests antigen spreading can lead to effective tumor control. However, it also points to the need to evaluate factors that render the protection incomplete. In the B16/OVA model, we used mTRP1 and mTRP2 as surrogate markers for antigen spreading. The implication is that through treatment-induced antigen spreading, T cells against other B16/OVA tumor antigens may have been generated (i.e., mTRP1 and mTRP2 may just be two of the many), and the collective of these tumor-specific T cells would be able to eliminate recurring tumor cells, even if the tumor cells have downregulated vaccine-targeted antigen(s). In the survivor mice, T cell responses against mTRP1 were low compared to responses against mTRP2 (Supplementary Fig. 7), demonstrating antigen spreading may generate a spectrum of low to high frequency of tumor-specific T cells, which may be one factor dictating effectiveness of recurrent tumor control. Our findings support the established strategy of targeting multiple tumor antigens to achieve optimal anti-tumor efficacy and suggest combination immunotherapies designed to induce antigen spreading may be an additional approach, especially in cases where only a few targetable tumor antigens have been identified.

Good prognosis has been associated with the presence of CD8 T cells^35–38^ or an increased ratio of CD8 T cells to regulatory T cells (CD8:Treg) in the tumor^39–42^. In our model, we compared the ratio of antigen-specific CD8 T cells to regulatory T cells in tumors of mice treated with ZVex alone or the G100–ZVex combination and determined there was no significant difference (Supplementary Fig. 5c). However, there was at least a doubling of percent lymphocytes in tumors from mice treated with the G100–ZVex combination (Supplementary Fig. 5a), suggesting, at least in this case, the overall increase in antigen-specific CD8 T cells was sufficient to overcome a stagnant CD8:Treg ratio and achieve significant anti-tumor control. Furthermore, T cell repertoire within the TME of mice treated with the G100–ZVex combination had decreased clonality (and thereby increased diversity) (Supplementary Fig. 6), likely as a result of antigen spreading induced by the combination, which could potentially be used to predict and/or assess patient response to immunotherapies. For example, the lack of a strong pre-treatment TCR clonality suggests a non-T cell-inflamed TME that may need interventions to induce not just a more inflammatory TME but also tumor-specific T cells. Similarly, increased post-treatment TCR diversity may suggest antigen spreading, a sign that the current regimen may be working. We also compared the ratio of CD8 T cells to CD4 T cells (CD8:CD4) in tumors of mice treated with ACT alone or the G100–ACT combination (Fig. 4f–h). Following the classic prognostic correlation with therapeutic response, the G100–ACT combination best increased percent CD8 T cells and the CD8:CD4 ratio in the TME, compared to untreated or monotherapy-treated mice. Strikingly, tumors of mice treated with the G100–ACT combination had drastically lower percentage of CD8^−^ CD4^−^ cells (Fig. 4f), a population of cells that have been previously identified as “defective tumor-associated T cells,” whose presence is associated with tumor progression^43^. In sum, evaluation of prognostic biomarkers should continue to consider population shifts between CD8 and CD4 or regulatory T cells (or even potentially defective tumor-associated T cells) but could perhaps include evaluation of relative or absolute changes in tumor-infiltrating antigen-specific T cells and/or T cell repertoire changes within tumors pre- and post-treatment.

The ultimate goal of cancer immunotherapy is to induce tumor-specific immune responses that could eliminate existing tumor cells (most likely post-surgical debulking) and prevent recurrence. We have demonstrated that the anti-tumor efficacy achieved with the G100–ZVex combination was mediated largely by CD8 T cells, induced directly by ZVex immunization and indirectly via antigen spreading. ZVex combined with G100 was key to drastically shift the TME to a more inflamed milieu, thereby promoting immune cell functions that led to the complete eradication of existing tumor mass and generation of tumor-specific immunological memory that prevented recurrence. We show here that established B16 tumors can be completely eradicated using a combination of systemic and in situ immunizations, a potentially effective strategy to convert non-T cell-inflamed tumors to T cell-inflamed tumors.

## Methods

### Vector production and quantitation

ZVex/OVA vectors were designed and produced based on the VP02 platform previously described^44^. Briefly, vectors were produced via the transient transfection of 293T cells with 5 plasmids: the transfer vector that encodes the VP02 genome and ovalbumin, a modified gagpol transcript (RI-gagpol), accessory protein Rev from HIV-1, accessory protein Vpx from SIVmac, and the E1001 envelop glycoprotein variant of Sindbis virus. To quantify each vector lot, genomic RNA was isolated from vector particles using the QIAamp Viral RNA Mini kit (Qiagen, Valencia, CA). To eliminate contaminating DNA, the extracted nucleic acid was digested with DNase I (Invitrogen, Grand Island, NY). Two dilutions of each DNase I-treated RNA sample were then analyzed by quantitative RT-PCR using the RNA Ultrasense One-Step Quantitative RT-PCR System (Invitrogen) and vector-specific primers and probe (Integrated DNA Technologies, Coralville, Iowa):

Forward primer: 5′-GGCAAGCAGGGAGCTAGAAC-3′

Reverse primer: 5′-GTTGTAGCTGTCCCAGTATTTGTC-3′

Probe: 5′-(FAM)-TCGCAGTTAATCCTGGCCTGTTAGA-(BHQ)-3′

The vector RNA copy number was calculated in reference to a standard curve comprised of linearized plasmid DNA containing the target sequences, diluted over a 7-log range (1 × 10^7^ to 1 × 10^1^ copies). The genome titer used throughout the experiments reflects the number of physical vector particles, calculated based on genomes, with each vector particle predicted to contain 2 single-stranded copies of genomic RNA.

### Murine tumor models and cell lines

Female 7–8 week-old C57BL/6, OT-I, PMEL, and B6.SJL mice were obtained from the Jackson Laboratory (Bar Harbor, ME) or Taconic Biosciences (Rensselaer, NY). Mice were housed under specific pathogen-reduced conditions in a BSL-2^+^ level room in the Infectious Disease Research Institute (IDRI) animal facility (Seattle, WA).

B16F10 cells were purchased from ATCC (Manassas, VA). To generate B16F10 cells expressing ovalbumin, B16F10 cells were transduced with lentiviral vector encoding ovalbumin. Transduced cells were serially diluted to near single-cell density per well of a 96-well plate for clonal expansion. Each clone was stained with anti-ovalbumin antibody (Thermo Fisher Scientific, San Francisco, CA) to confirm expression of ovalbumin by flow cytometry analysis. Clones expressing high or low protein levels of ovalbumin were inoculated subcutaneously into flanks of C56BL/6 mice for evaluation of tumor take. Clone 11 (referred to as “B16/OVA” throughout this report) was selected for its uniform expression of ovalbumin and reproducible induction of tumor growth when inoculated subcutaneously into flanks of mice. B16/OVA cells were maintained in complete RPMI media (i.e., RPMI 1640 supplemented with 10% fetal bovine sera, 1 mM sodium pyruvate, 55 μM 2-mercaptoethanol, 1× MEM non-essential amino acids, and 1× penicillin/streptomycin/glutamine; all from Thermo Fisher Scientific, San Francisco, CA).

GL261 cells expressing ovalbumin and luciferase (referred to as “GL261/OVA” throughout this report) were a kind gift from Dr. Aaron J. Johnson (Mayo Clinic, Rochester, MN)^45^. GL261/OVA cells were maintained in complete DMEM (i.e., DMEM media supplemented with 10% fetal bovine sera, 4.5 g/L D-glucose, L-glutamine; all from Thermo Fisher Scientific).

On the day of tumor inoculation, B16/OVA or GL261/OVA cells in logarithmic growth were resuspended in HBSS at 1 × 10^5^ cells/40 μl or 2 × 0^5^ cells/2 μl, respectively, and transported on ice to the animal facility for injections.

For inoculation of B16/OVA cells, fur at the inoculation site was shaved after each mouse was anesthetized, followed by inoculation of the B16/OVA cells via a 40 μl subcutaneous injection into the right flank using a 29G 0.3 ml insulin syringe (BD Biosciences, San Jose, CA). Tumor growth was assessed two to three times a week by caliper measurement of tumor length and width. Tumor volumes were calculated using a modified ellipsoid formula: length × width^2^ × π/6. Mice were euthanized when tumor volume exceeded 1200 mm^3^.

For orthotopic inoculation of GL261/OVA cells, hair on heads of mice were removed with Nair (Church & Dwight, Ewing, NJ) the day before inoculation surgery. On the day of surgery, a burr hole in the skull of each anesthetized mouse was drilled at the injection coordinates stereotactically marked by 2.5 mm lateral and 0.5 mm anterior of bregma. Each mouse was then inoculated with GL261/OVA cells via a 2 μl intracranial injection, 3 mm deep from the cortical surface, into the left striatum, at a rate of 0.2 μl/min. Tumors were visualized 10–15 min after intraperitoneal injection of D-luciferin (Perkin Elmer, Waltham, MA) at 150 mg/kg. Mice were euthanized when body weight loss exceeded 20%.

Animals were monitored daily for survival. All procedures were approved by the IDRI Institutional Animal Care and Use Committee, and all efforts were made to minimize suffering of the mice.

### Therapeutic treatments

Our previous experience (unpublished observations) showed that therapies (mono- or combination) that start when tumors are over 7 mm in diameter (average 100–150 mm^3^) are likely ineffective—or, at best, can only achieve delayed tumor growth. To eradicate a tumor, either the tumor needs to be surgically removed (which remains first line therapy for established solid tumors in humans but beyond the scope of this report and thus not modeled here), or treatments need to start when tumors are smaller. The largest average tumor size we have been able to demonstrate eradication of treated tumors (without surgical intervention) is 50–80 mm^3^. For all studies reported within, we thus initiated treatments once tumors were established, defined by measuring ≥5 mm in tumor diameter, with tumor volume averaging 50–80 mm^3^.

Aliquots of ZVex vectors stored at −80 °C were thawed at room temperature and then kept on ice. Vectors were diluted in cold sterile HBSS to the desired concentration and transported to the animal facility on ice for injections. Mice were placed in a conventional slotted restrainer with the tail base accessible. Vector was administered once via a 50 μl injection using a 29G 0.3 ml insulin syringe (BD Biosciences, San Jose, CA) inserted subcutaneously at the tail base, ~1 cm caudal to the anus, leading to minor but notable distension of the skin around the tail base. Vehicle control administered to control mice was HBSS.

GLA in 2% oil-in-water stable emulsion (SE) was formulated by Infectious Disease Research Institute (Seattle, WA), and its structure and molecular characteristics have been previously described^46^. Intratumoral administration of GLA (i.e., G100) was given twice weekly via a 50 μl injection using a 29G 0.3 ml insulin syringe (BD Biosciences) inserted into the tumor, at a dose of 5 μg per 2% SE. G100 was administered intratumorally every 2–3 days until tumors completely regressed or until mice were sacrificed due to tumor burden. The 5 μg dose was chosen based on unpublished observations that 5–10 μg doses induced the best adjuvant effect, with lower and higher doses resulting in loss of adjuvant effect. The dosing regimen (i.e., q2–3d) was chosen based on pilot studies where we evaluated G100 regimen (at the 5 μg dose) at q1d, q2–3d, and q7d, and we observed the best therapeutic effect at q2–3d. When dosed at q1d, we observed no additional therapeutic benefit. Vehicle control administered to control mice was 2% SE.

Adoptive cell transfers were generated from transgenic mice that are genetically capable of educating CD8+ T cells against a specific antigen (e.g. SIINFEKL epitope of ovalbumin protein). Transgenic donor mice (OT-I or PMEL, The Jackson Laboratory) were immunized approximately 2 weeks prior to their spleens being harvested for adoptive transfer studies. Splenocytes were then processed, and magnetic activated sorting kits (Miltenyi) were used to isolate CD8^+^ T cell populations. Sorted CD8^+^ T cells (2 × 10^6^–10 × 10^6^ cells per transfer) were resuspended in HBSS and transferred into tumor-bearing recipient mice intravenously via retro-orbital injection once flank tumors were palpable.

### Antibody depletion

Mice were administered 100 μg/dose of anti-CD8 (clone 2.43, Bio X Cell, West Lebanon, NH), anti-CD4 (clone GK1.5, Bio X Cell), anti-NK1.1 (clone PK136, Bio X Cell), or the corresponding isotype control antibody, intraperitoneally, on the day of tumor challenge. Depletion antibodies were continuously administered every 3–4 days until the end of the study. To confirm successful depletion (>95%) of the target population, blood from mice was collected, stained for B220 (clone RA3-6B2, BD Biosciences), CD3ε (clone 145–2C11, Thermo Fisher Scientific), CD8 (clone H35–17.2, Thermo Fisher Scientific), CD4 (RM4–5, Thermo Fisher Scientific), and NKp46 (29A1.4, Thermo Fisher Scientific), and analyzed by flow cytometry.

### Flow cytometry analysis

Spleens and tumors were homogenized with gentleMACS Octo Dissociator (Miltenyi Biotec), per the manufacturer’s protocol. Red blood cells were lysed with 1× RBC Lysis Buffer (BioLegend, San Diego, CA), per manufacturer’s protocol. For staining with dextramers or tetramers (collectively referred to as “multimers” throughout this report), cells were incubated with H-2Kb-SIINFEKL-PE (OVA_257–264_), H-2Db-TAPDNLGYM-PE (mTRP1_455-A463M_), H-2Kb-SVYDEFVWL-APC (mTRP2_180–188_) (all from Immudex, Copenhagen, Denmark), or H-2Db-EGSRNQDWL-APC (m-gp100_25–33_) (MBL International Corporation, Woburn, MA) for 10 min at room temperature, followed by surface staining and fixation (detailed below). For analysis of cytokines, cells were stimulated in 96-well round-bottom plates with peptides at a concentration of 1 μg/ml per peptide in complete RPMI media for 5 h at 37 °C, 5% CO_2_ in the presence of brefeldin A (GolgiPlug, BD Biosciences, San Jose, CA). Peptides, including OVA_257–264_, mTRP1_455-A463M_, mTRP2_180–188_, and gp100_25–33_, were manufactured at 95% purity by New England Peptide (Gardner, MA). After peptide stimulation, surface staining was carried out in flow cytometry staining (FCS) buffer (PBS, 1% FCS, 2 mM EDTA) in the presence of FcR blocking antibody 2.4G2 and LIVE/DEAD Fixable Near-IR (L/D NIR). Antibodies used for surface staining included anti-mouse CD3ε-PerCP/Cy5.5, CD4-Alexa Fluor 700, CD8-eFluor 450, and B220-V500 (BD Biosciences). After surface staining, cells were washed with FCS buffer, fixed with Cytofix (BD Biosciences), and stored overnight at 4 °C in FCS buffer. Cells were then permeabilized with Perm/Wash buffer (BD Biosciences) containing 5% rat serum (Sigma-Aldrich, St. Louis, MO). Antibodies for intracellular staining were diluted in Perm/Wash buffer containing 5% rat serum and added to permeabilized cells. Antibodies included anti-mouse TNF-FITC, IFNγ-PE, and IL-2-APC. Cells were washed with Perm/ Wash buffer, resuspended in FACS buffer, and analyzed on a 3-laser LSRFortessa with High Throughput Sampler (BD Biosciences). Data were analyzed using FlowJo software (Tree Star, Ashland, OR). Viable CD8 T cells were gated as follows: lymphocytes (FSCint, SSClo), single cells (SSC-A = SSC-H), live (L/D NIR^lo^), CD3ε^+^ B220^−^, CD4^−^ CD8^+^. Cytokine gates were based on the 99.9th percentile (<0.1% of positive events in non-stimulated cells). All materials, otherwise noted, were purchased from Thermo Fisher Scientific.

### T cell receptor (TCR) sequencing and analysis

For TCR repertoire analysis, total genomic DNA was extracted from whole tumors using the spin column method and the DNeasy kit (Qiagen). High-throughput deep sequencing was used to analyze the TCRβ CDR3 region with the Illumina Genome Analyzer (Adaptive Biotechnologies, Seattle, WA) using the immunoSEQ immune-profiling system^47^. In-frame unique sequences without stop codons, referred to as unique productive sequences, were used for the repertoire analysis. Identification of the Vβ, Dβ, and Jβ gene segments contributing to each TCRβ CDR3 sequence was performed using the published algorithm. To determine T cell receptor clonality of tumor samples, Shannon entropy was calculated on the estimated number of genomes of all productive TCRs and normalized by dividing by the log_2_ of unique productive sequences in each sample. Clonality was calculated as 1- normalized entropy. We computationally identified clones with significantly different abundances between two samples using a binomial test with Benjamini–Hochberg corrected p-values, such that false-discovery-rates were held at 5% ^48^.

### Gene expression analysis

Total RNA was isolated using the AllPrep kit (Qiagen, Valencia, CA) from freshly harvested tumors. RNA concentration was quantified using UV spectroscopy with a Nanodrop device (NanoDrop Products, Wilmington, DE). A total of 200 ng RNA was used for gene expression analysis using the human nCounter PanCancer Immune Profiling Panel (Nanostring Technologies, Seattle, WA), which includes 770 genes. Sample preparation and the hybridization was carried out using the nCounter Preparation Station according to the manufacturer’s instructions. Data were collected using the nCounter Digital Analyzer and data normalization and analysis were carried out using the nSolver software.

### Statistical analysis

Mann–Whitney or ANOVA tests were used to calculate statistical significance, where appropriate. All statistical analyses were calculated using the Prism 8.0 application program (GraphPad, La Jolla, CA).

### Ethical considerations

All mice were maintained in a pathogen-free animal facility at the Infectious Disease Research Institute (IDRI, Seattle, WA), according to its institutional guidelines. All animal protocols used in this study adhere to the Institutional Animal Care and Use Committee (IACUC) guidelines and were approved by the IDRI IACUC committee, protocol number 2015-14. The protocol under which these studies were conducted was originally approved on September 30, 2015 and has been reviewed and approved every 12 months, with a formal renewal every 3 years. The most recent renewal (protocol number 2018-17) was approved on October 12, 2018, with an approved review on August 15, 2019.

### Reporting summary

Further information on research design is available in the Nature Research Reporting Summary linked to this article.

## Supplementary information

Reporting Summary

Supplementary Information

## Data Availability

We confirm that the data supporting the findings of this study are available within the article and its Supplementary material.
