# Supplementary material for: Intratumoral immune activation with TLR4 agonist synergizes with effector T cells to eradicate established murine tumors
Source: NPJ Vaccines. 2020 Jun 16;5:50. doi: 10.1038/s41541-020-0201-x (PMC7298055; doi:10.1038/s41541-020-0201-x)
Supplement: Supplementary file 2 — Supplementary Information [file 41541_2020_201_MOESM2_ESM.pdf]

Supplementary Figure 1.

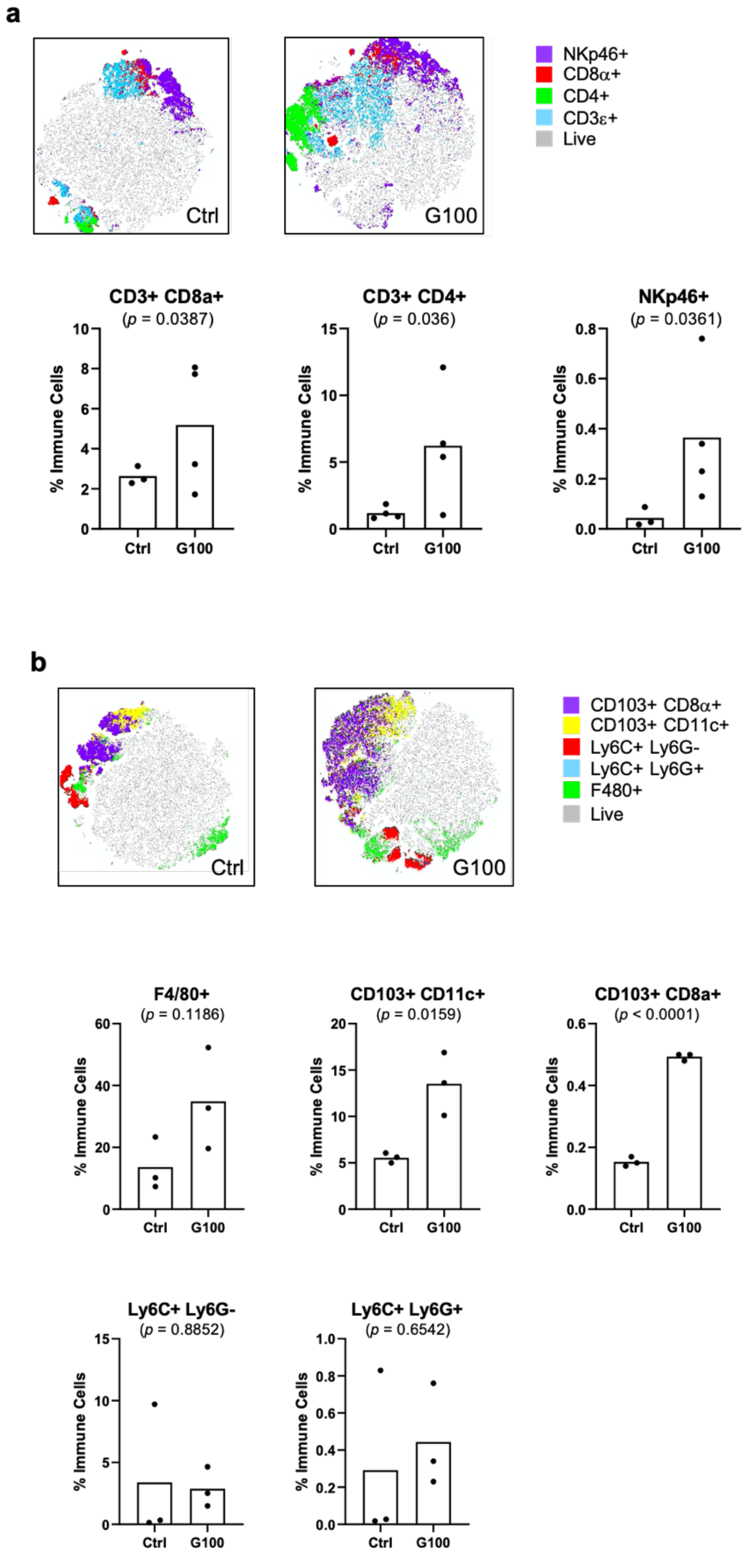

Supplementary Figure 1. G100 increases immune cell infiltrates within the TME. Female C57BL/6 mice (n = 3-5/group) were inoculated with B16/OVA cells (flank, subcutaneously) on Day 0. Once tumors became palpable (averaged 5 mm in diameter), tumor-bearing mice were given two intratumoral G100 administered 3-4 days apart. Tumors were harvested 24 hours post-last G100 administration, processed to single cell suspensions, stained for **(a)** lymphocytes or **(b)** myeloid cells, analyzed by flow cytometry, and visualized with tSNE plots (top panels). Flow cytometry data per mouse from each group were concatenated prior to tSNE analysis. Defined cell populations labeled in the figure were also quantified and presented as bar graphs (bottom panels). Data are representative of at least 2 independent experiments.

Supplementary Figure 2.

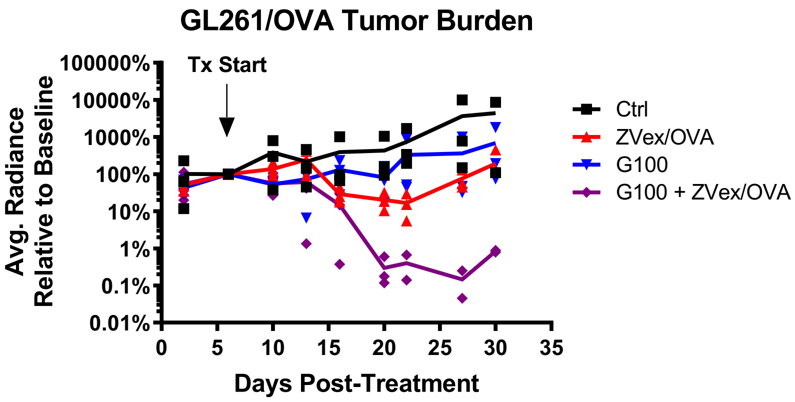

Supplementary Figure 2. G100-ZVex combination eradicates orthotopic glioblastomas. In an orthotopic glioblastoma model, female C57BL/6 mice (n = 3/group) were cannulated about a week prior to being stereotactically inoculated with GL261/OVA cells expressing luciferase, intracranially. Tumor burden was determined by intensity of luciferase activity, measured by IVIS imaging system. Once tumors averaged > 10^5 radiance, mice were immunized once with ZVex/OVA and given intratumoral G100 that was continued once weekly for a maximum of 3 doses. Mice were sacrificed at > 20% weight-loss. All tumor-bearing mice were followed until they became tumor-free or were euthanized due to tumor burden. Average percent change in radiance, relative to radiance measured on start date of treatment (6 days post-tumor inoculation), is graphed. Data are representative of at least 3 independent experiments. (Statistics were not calculated due to low sample size.)

Supplementary Figure 3

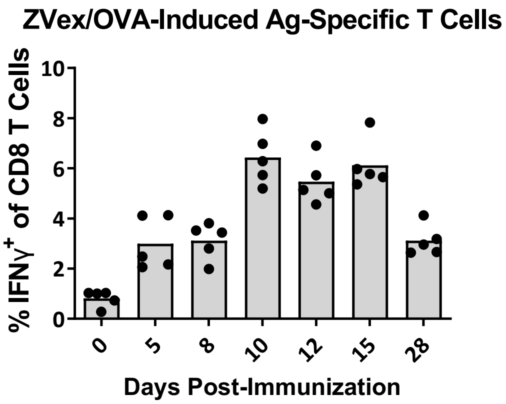

Supplementary Figure 3. ZVex/OVA induces OVA-specific CD8 T cells. Female C57BL/6 mice (n = 5/group) were immunized once with ZVex/OVA. Individual splenic T cell responses were measured by intracellular cytokine staining and plotted at the time points listed in the figure. Data are representative of at least 3 independent experiments.

Supplementary Figure 4

a

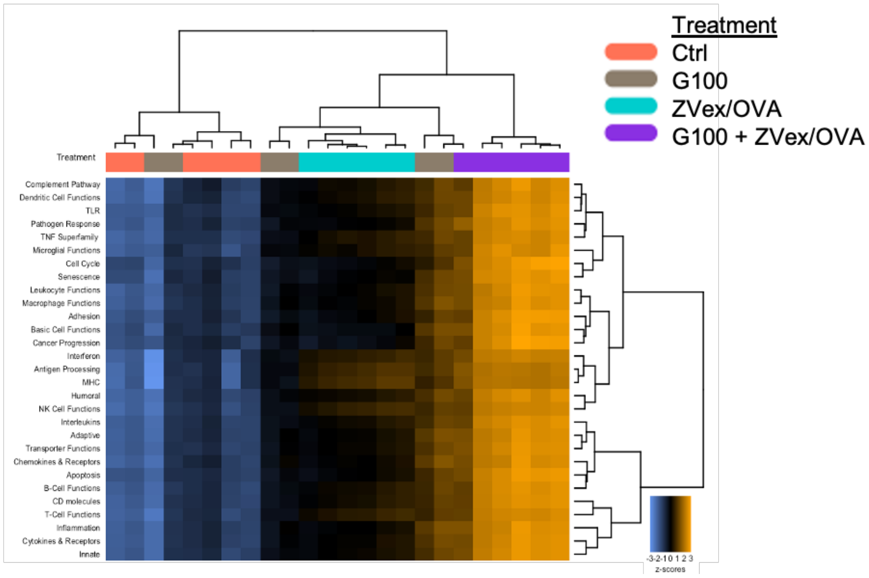

b

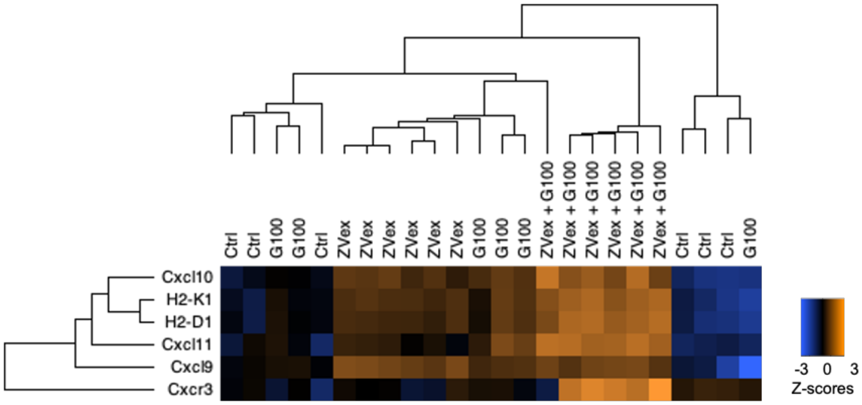

Supplementary Figure 4. G100-ZVex combination promotes a T cell-inflamed TME. Female C57BL/6 mice (n = 3/group) were inoculated with B16/OVA cells (flank, subcutaneously) on Day 0. Once tumors

became palpable (averaged 5 mm in diameter), mice were immunized once with ZVex/OVA and given two intratumoral G100 administered 3-4 days apart. 24 hours post-last G100 administration, RNA isolated from tumors of mice from each treatment group was analyzed for expression, using the PanCancer Immune Profiling Panel from Nanostring Technologies. **(a)** Pathway signatures or **(b)** selected individual gene expressions are presented as heatmaps. Data are representative of at least 2 independent experiments.

Supplementary Figure 5

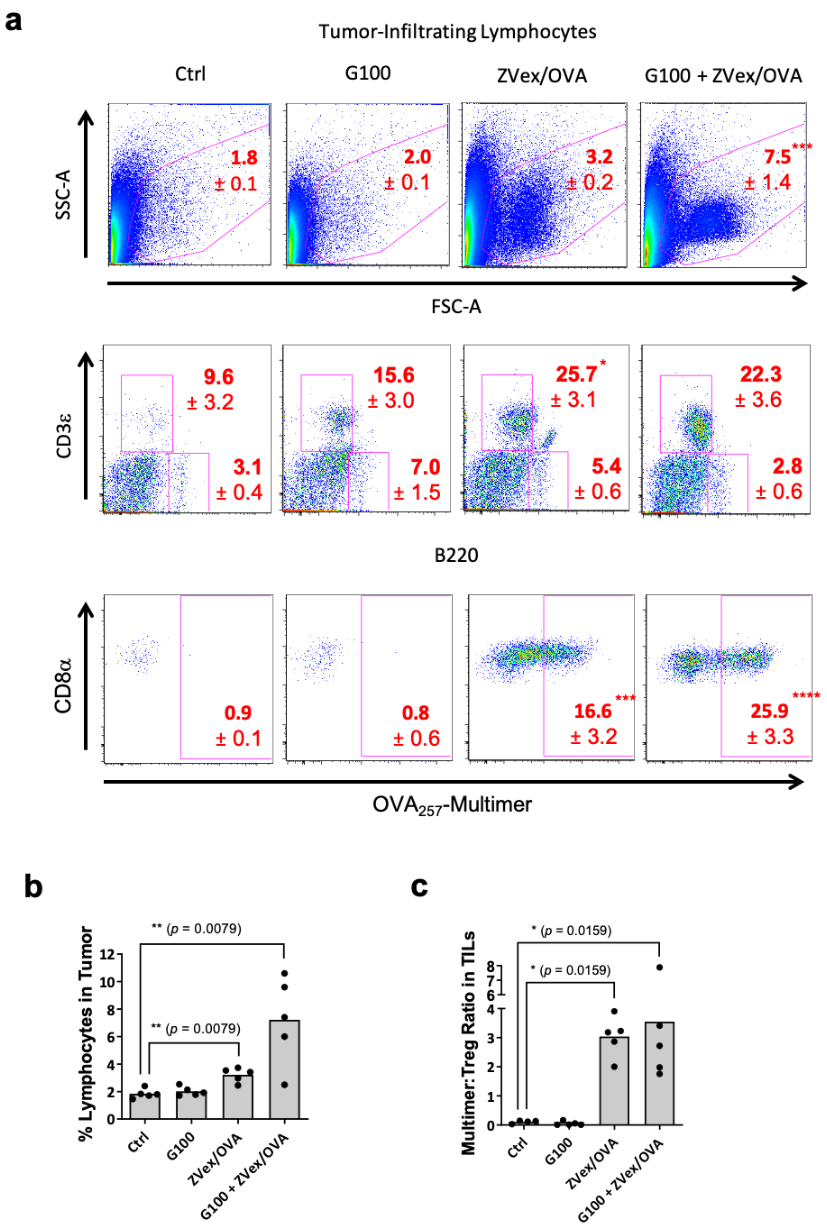

Supplementary Figure 5. G100 improves tumor infiltration of ZVex-induced tumor-specific CD8 T cells. Female C57BL/6 mice (n = 5/group) were inoculated with B16/OVA cells (flank, subcutaneously) on Day 0. Once tumors became palpable (averaged 5 mm in diameter), mice were immunized once with ZVex/OVA and given two intratumoral G100 administered 3-4 days apart. Tumors were harvested 24 hours post-last G100 administration, processed to single cell suspensions, stained with the markers

listed, and analyzed via flow cytometry: **(a)** Representative flow cytometry diagrams, with percent averages ± SEM of gated populations indicated; **(b)** percent lymphocytes (individually plotted) of total tumor-infiltrating lymphocytes; **(c)** ratio of OVA-specific CD8 T cells to regulatory T cells (individually plotted). Gating strategy: Singlets (SSC-A = SSC-H), lymphocytes (FSC-A vs. SSC-A), live cells (fixable live/dead stain negative), T cells ($CD3^+$ $B220^-$), CD8 T cells ($CD8^+$ $CD4^-$), OVA-specific CD8 T cells ($multimer^+$ $CD8^+$). Data are representative of at least 3 independent experiments. (* $p < 0.05$, ** $p < 0.01$, *** $p < 0.001$, **** $p < 0.0001$)

Supplementary Figure 6

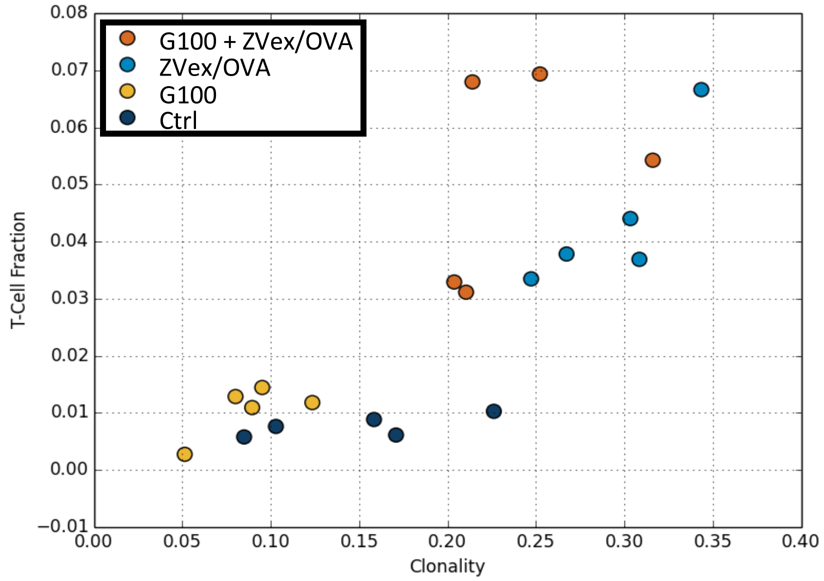

Supplementary Figure 6. G100-ZVex combination increases T cell fraction and TCR diversity within the TME. Female C57BL/6 mice (n = 5/group) were inoculated with B16/OVA cells (flank, subcutaneously) on Day 0. Once tumors became palpable (averaged 5 mm in diameter), mice were immunized once with ZVex/OVA and given two intratumoral G100 administered 3-4 days apart. Tumors were harvested 24 hours post-last G100 administration and sent to Adaptive Biotechnologies to define the T cell repertoire within the tumor microenvironment via T cell receptor sequencing. Data are representative of at least 2 independent experiments.

Supplementary Figure 7

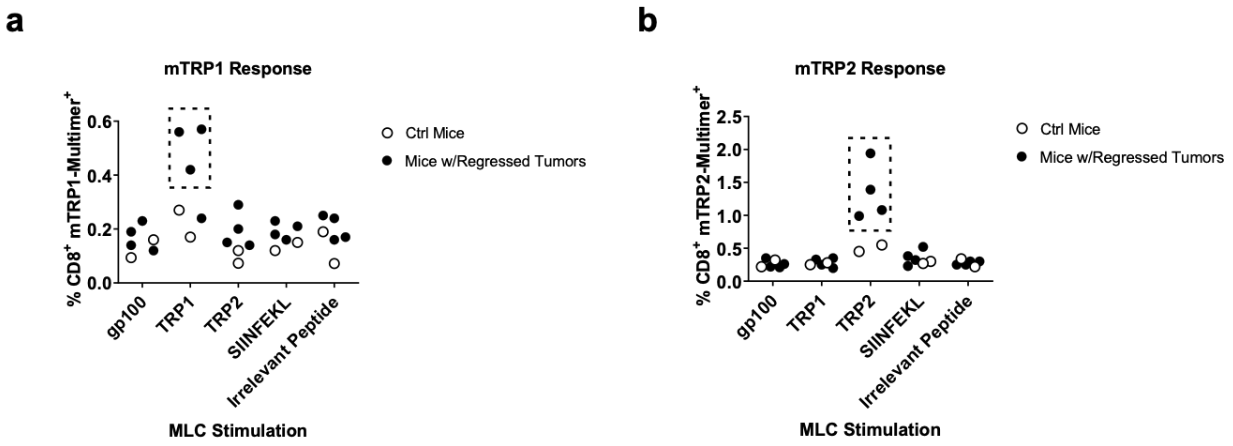

Supplementary Figure 7. G100-ZVex combination induces antigen spreading. Splenocytes isolated from age-matched naïve ("Ctrl") mice and tumor-bearing mice treated with the G100-ZVex combination that had complete tumor regression were incubated with mitomycin C-treated feeder cells pulsed with peptides listed in the figure for 5 days, followed by multimer staining for **(a)** mTRP1- and **(b)** mTRP2-specific T cells. Individual percent multimer$^+$ CD8 T cells of total CD3 T cells per mouse per group are graphed. Gating strategy: Singlets (SSC-A = SSC-H), lymphocytes (FSC-A vs. SSC-A), live cells (fixable live/dead stain negative), T cells (CD3$^+$ B220$^-$), CD8 T cells (CD8$^+$ CD4$^-$), OVA-specific CD8 T cells (CD8$^+$ multimer$^+$). Data are representative of at least 2 independent experiments.

Supplementary Figure 8

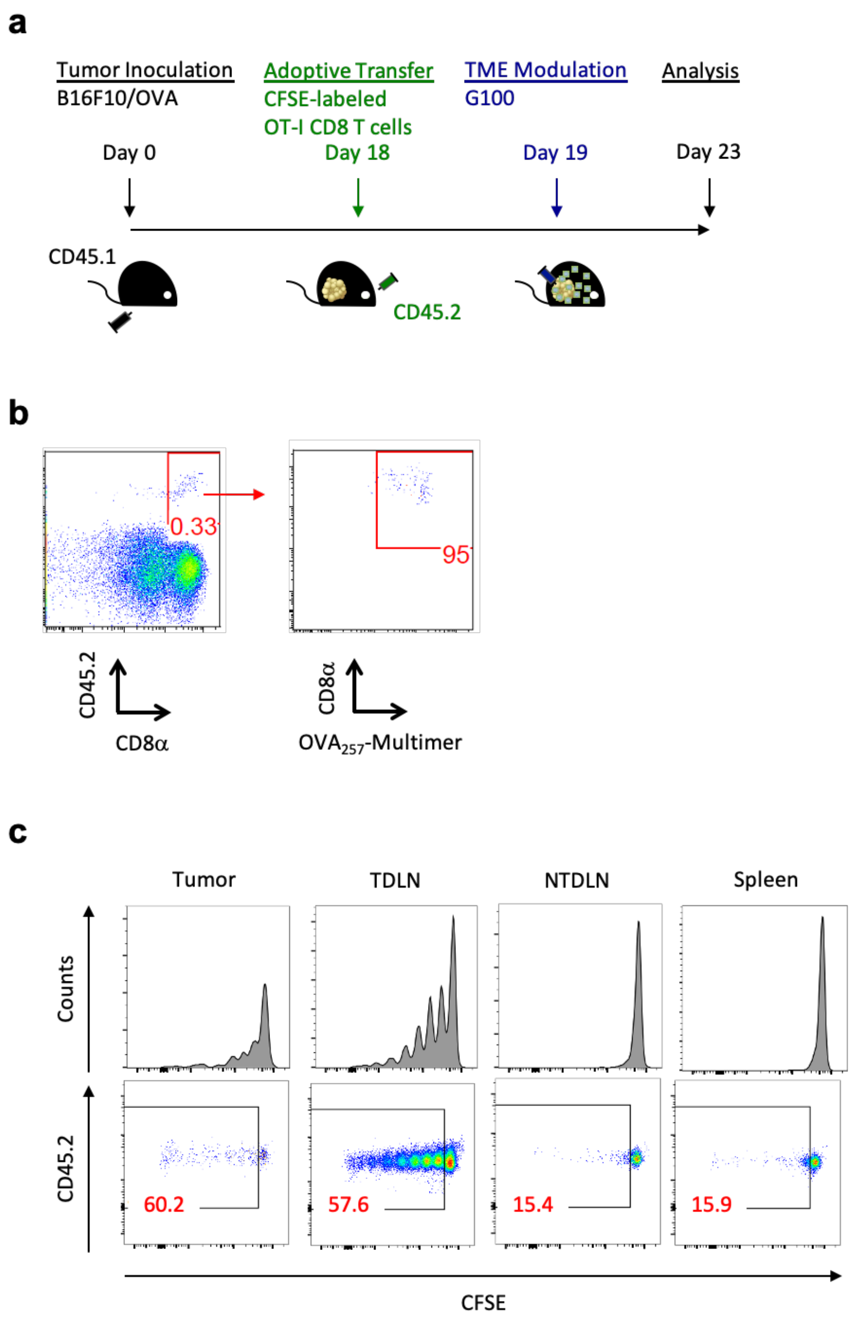

Supplementary Figure 8. Adoptively transferred T cells proliferate within G100-treated tumors. (a) Schematic of tumor inoculation, adoptive transfer, and treatment, to track trafficking of OVA-specific CD8 T cells post-G100 modulation of the TME. On Day 0, female B6.SJL mice ($CD45.1^+$, n = 10/group) were inoculated with B16/OVA cells (flank, subcutaneously). When tumors reached 50 mm^3, tumor-bearing B6.SJL mice were administered intratumoral 5 μg G100. G100 was dosed continually every 3-4 days until end of study. On Day 18, 1×10^7 CFSE-labeled OT-I CD8 T cells ($CD45.2^+$) were adoptively transferred to each tumor-bearing B6.SJL mice, followed by one intratumoral injection of G100 on Day 19. On Day 23, lymphocytes were isolated from harvested spleens, tumors, tumor draining and non-draining lymph nodes, and stained for flow cytometry analysis: Representative flow cytometry diagrams of **(b)** splenocyte samples and **(c)** listed samples, with percent averages of gated populations indicated. Gating strategy: **(b)** Singlets (SSC-A = SSC-H), lymphocytes (FSC-A vs. SSC-A), live cells (fixable live/dead stain negative), T cells ($CD3^+$ $B220^-$), transferred CD8 T cells ($CD45.2^+$ $CD8^+$), OVA-specific transferred CD8 T cells ($CD8^+$ $multimer^+$); **(c)** singlets (SSC-A = SSC-H), lymphocytes (FSC-A vs. SSC-A), live cells (fixable live/dead stain negative), T cells ($CD3^+$ $B220^-$), transferred proliferating cells ($CD45.2^+$ $CFSE^+$). Data are representative of at least 3 independent experiments.
